# Role of native Thiol, total Thiol and dynamic Disulphide in diagnosis of patient with prostate cancer and prostatitis

**DOI:** 10.1590/S1677-5538.IBJU.2018.0469

**Published:** 2019-07-27

**Authors:** Mehmet Solakhan, Hülya Çiçek, Nuri Orhan, Mustafa Yildirim

**Affiliations:** 1 Department of Urology, Medicalpark Gaziantep Hospital, Bahçeşehir University School of Medicine, Gaziantep, Turkey;; 2 Department of Medical Biochemistry, Medicalpark Gaziantep Hospital, Gaziantep, Turkey;; 3 Department of Internal Medicine, Medicalpark Gaziantep Hospital, Bahçeşehir University School of Medicine, Gaziantep, Turkey;; 4 Department Medical Oncology, Medicalpark Gaziantep Hospital, Bahçeşehir University School of Medicine, Gaziantep, Turkey

**Keywords:** Prostatic Neoplasms, Oxidative Stress

## Abstract

**Background:**

Our study investigates whether Native Thiol, Total Thiol and disulphide levels measured in serum of patients with prostate cancer and prostatitis and of healthy subjects, have any role in differential diagnosis.

**Materials and Methods:**

Patients followed up for histopathologically verified diagnosis of prostate cancer and prostatitis in 2016-2017 at the Medicalpark Gaziantep Hospital Urology Clinic were included in the study. Native Thiol (NT), Total Thiol (TT), Dynamic Disulphide (DD) levels in serum were measured by a novel automated method.

**Results:**

NT, TT, DD, NT / TT ratios, DD / TT ratio and DD / NT ratio were measured as 118.4 ± 36.8μmoL / L, 150.3 ± 45.3μmoL / L, 15.9 ± 7μmoL / L, 78.8 ± 7μmoL / L, 10.5 ± 3.5μmoL / L, 13.8 ± 5.8μmoL / L respectively in patients with prostate cancer; as 116.4 ± 40.5μmoL / L, 147.5 ± 50.1μmoL / L, 15.5 ± 8.7μmoL / L, 79.7 ± 9μmoL / L, 10.1 ± 4.5μmoL / L, 13.5 ± 7.2μmoL / L in patients with prostatitis and as 144.1 ± 21.2μmoL / L, 191 ± 32.3μmoL / L, 23.4 ± 10.1μmoL / L, 76.1 ± 98.3μmoL / L, 11.9 ± 4.1μmoL / L, 16.4 ± 6.9μmoL / L in healthy subjects. Significant difference was detected between groups of NT, TT and DD levels (p = 0.008, p = 0.001, p = 0.002). No significant difference was detected in terms of the NT / TT, DD / TT and DD / NT rates (p = 0.222, p = 0.222, p = 0.222).

**Conclusions:**

Serum NT, TT, DD levels in patients with prostatitis and prostate cancer were found significantly lower compared to the control group. This indicates that just as inflammation, prostate cancer also increases oxidative stress on tissues.

## INTRODUCTION

Prostate cancer is the second most prevalent type of cancer in men and is the second most prevalent cause of death from cancer in men. Past studies have shown the important role of age, genetic predisposition, androgen hormones, diet-related factors, inflammation and oxidative stress in the development of this disease (1). It was reported that oxidative stress can play a significant role in the development of prostate cancer through lipid per-oxidation and similar mechanisms (2).

It was reported that oxidative stress resulted from the disruption of the balance of antioxidants and reactive oxygen radicals and that this caused various systemic diseases. Over-production of reactive oxygen types (ROT) causes damage in proteins and lipids. Oxidative damage is irreversible in serum and tissue proteins and significant changes occur in the structure and activity of proteins and biomolecules. Oxidative modifications in DNA and proteins can impact certain cellular functions, resulting in cell damage, death or mutation and carcinogenesis formation (2).

Thiols are essential and strong anti-oxidant molecules in the sulfhydryl group, consisting of hydrogen atom and sulfur atom bonded to a carbon atom (3). The disulphide bond in their structure is a covalent bond and is also named as SS-bond or disulphide bridge. They play an important role in protecting oxidant stress from harmful effects. The leading thiols found in plasma are low molecule-weight thiols including albumin thiols, protein thiols and cysteine, cysteinylglycine, glutathione, homocysteine and γ-glutamylcysteine. The thiol groups are oxidized with disulphide bonds getting reversibly oxidized by ROTs. This mechanism mediates their anti-oxidant effects (4). The created disulphide bonds can again be reduced to thiol groups. Dynamic thiol-disulphide homeostasis plays an important role in anti-oxidant defense, detoxification, apoptosis, arranging enzymatic activity and cellular signal transmission (5).

When oxidative stress occurs, it has been noted than reduced thiol concentration increases and disulphide values increase in correlation (6). Studies have reported this deterioration of homeostasis leads to chronic kidney deficiency, diabetes mellitus, cardiovascular diseases, cancer, chronic inflammatory diseases and various neuro-degenerative diseases (4).

Prostate Specific Antigen (PSA) is a critical marker in prostate cancer diagnosis. Serum total prostate specific antigen (t PSA) levels together with abnormal digital rectal examination were the most prevalent methods used in prostate biopsy indication in recent years (7). Increased levels of serum PSA are associated not only with cancer but also with bacterial prostatitis, prostatic inflammation, benign prostate hypertrophy and urinary system infection (8).

Prostatitis is a disease that is observed at a rate of 8.2% (2.2-9.7%) in men. Acute bacterial prostatitis (ABP) is a pyogenic urinary system infection of the urinary system. It is observed at a rate of 5% among general prostatitis (8). Escherichia coli is the most frequent cause of acute bacterial prostatitis. Enterococcus, Proteus, Pseudomonas, Klebsiella and Serratia are factors less frequently responsible. ABP can cause urinary retention causing edema in the prostate. It can also cause serious complications from prostate abscess to urosepsis (8). Its treatment is usually performed according to clinical symptoms. Parenteral antibiotics and hydration are performed in the early stage. Catheter and drainage are implemented if unable to urinate. Serum PSA values are usually high in ABP (9). Additionally, high levels of erythrocyte sedimentation rate (ESR), C-reactive protein (CRP) and white blood cell count in full blood count accompany the situation.

In our study, serum t PSA level, NT, TT, DD levels were measured in patients diagnosed with prostate cancer and in healthy subjects at the Medicalpark Gaziantep Hospital Urology Clinic and Disulphide / NT, Disulphide / TT, NT / TT ratios were calculated to find Dynamic Thiol / Disulphide homeostasis levels for the purpose of investigating whether it has predictive value in differentiating Prostatitis-Prostate Cancer and diagnosing Prostate Cancer and its value in predicting prognosis.

## MATERIAL AND METHODS

### Patient Selection

The study was conducted on patients histopathologically diagnosed with prostate cancer or prostatitis and followed up by the Gaziantep Medicalpark Hospital Urology Clinic in 2014-2017 and on healthy subjects compatible with the patients in terms of age. Patients previously treated and with metastasis, persons who were smoking and / or using alcohol, had chronic disease, acute or chronic infection or using antihyperlipidemic, antibiotic or anti-oxidant drugs were excluded from the study. We have taken informed consent form from the patients and healthy subjects to participate on the study. Socio-demographic characteristics, known diseases, medical history information such as personal and family history characteristics, used drugs etc., data such as routine laboratory tests were obtained retrospectively and recorded.

### Sampling and Measurement of NT, TT, DD

Blood samples of the patients were collected prior to starting the medication. Non-anticoagulant venous bloods of the subjects taken into tubes after 12 hours of fasting were centrifuged at 3500rpm for 10 minutes during the first 2 hours and then split into Eppendorf Microtubes and stored at −80°C. These samples were kept at 4°C temperature one night before measurement and put into room temperature 2 hours prior to the study and then the samples were mixed with a vortex and measurement was performed twice for each sample.

A novel automated assay method was used to measure dynamic thiol / disulphide homeostasis. The principle of this method is based on reducing the disulfide bonds of proteins to compose disulphides in oxidative medium. Sodium borohydride (NaBH4) is added for reduction of disulphide bonds into thiol groups again. The sum of residual thiol and reducted thiol groups encloses the total thiol. The remaining NaBH4 and DTNB (5,5’-dithio-bis-(2-nitrobenzoic acid)) are removed by formaldehyde. In this way, the amount of native and reduced thiol groups are defined separately. The difference between the TT and the NT is divided by two to calculate the quantity of the DD bonds. Also, we calculate the NT, TT, DD / TT, NT / TT and DD / NT percent ratio (10).

Measurement of serum t PSA levels were conducted automatically with the electro-chemiluminescent method using the Hitachi Modular Analytics E 170 device (Roche Diagnostics GmbH, Germany).

The patients were classified into risk groups according to Gleason score and t PSA values. Split into risk groups was as follows: low risk prostate cancer: T1-T2a stage and Gleason score ≤ 6 and t PSA ≤ 10, moderate risk prostate cancer: T2b stage and / or Gleason score = ≤ 7 and 10 ≤ t PSA ≤ 20 and high-risk prostate cancer: ≥ T2c stage or Gleason score 8-10 or t PSA > 20 (11).

### Statistical analysis

Statistical analyses were performed using the SPSS for Windows 15.0 package software. Compliance of the variables to normal distribution was examined using visual (histogram and probability graphs) and analytic methods (Kolmogorov-Smirnov / Shapiro-Wilk tests). In the Kolmogorov-Smirnov test, cases with p value greater than 0.05 were accepted as normal distribution. Differences between prostate cancer, prostatitis and the control groups in terms of NT, TT and DD were compared using the unilateral ANOVA test, as these variables showed normal distribution. The homogeneity of the variations was evaluated using the Levene test. Cases where the p value was lower than 0.05 were evaluated as statistically significant results. In cases with significant difference between the groups, post-hoc pair comparisons were performed using the Tukey's Test.

NT / TT, DD / TT and DD / NT ratios were detected to not show normal distribution. The differences between these variables between prostate cancer, prostatitis and control groups were compared using the Kruskal-Wallis test. Pair comparisons were performed using the Mann-Whitney U test and evaluated using the Bonferroni correction. Total type-1 error level was used as 5% for statistical significance.

## RESULTS

A total of 80 subjects were included in the study, consisting of 30 (37.5%) prostatitis patients, 25 (31.3%) prostate cancer patients and 25 (31.3%) healthy subjects. Patients diagnosed with prostatitis had a mean age of 60.5 ± 12.8 (range 31-83). Patients diagnosed with prostate cancer had a mean age of 70.6 ± 6 (range 58-82). The age difference between the two groups was evaluated using the Student t test as they had normal distribution. Statistically significant difference was detected between the two groups in terms of age (p = 0.001). Patients diagnosed with prostatitis consisted of younger patients.

Total Prostate Specific Antigen was detected as 139 ± 257.8 (range 4-1200) in patients with prostate cancer and as 51.3 ± 112 (range 3.9-405) in patients with prostatitis. Statistically significant difference was detected (p = 0.020) between patients with prostate cancer and prostatitis and tPSA value was detected to be higher in prostate cancer (Table-1).

**Table 1:** Comparison of ages and serum t PSA levels.

	Prostate Cancer (n:25)	Prostatitis (n:30)	Control Group (n:25)	p Value
Age(years)	70.6±6	60.5±12.8	64.3±25	0.001*
Mean±SD (min-max)	(58-82)	(31-83)	(61-76)	
tPSA(ng/mL)	139±257.8	51.3±112	1.2±4	0.001*
Mean±SD(min-max)	(4-1200)	(3.9-405)	(0.9-2.3)	

* p value is statistically significantly different

Prostate cancer patient group NT was detected as 118.4 ± 36.8 (range 68.1-201.3) μmoL / L, TT 150.3 ± 45.3 (range 87.2-243.9) μmoL / L, DD 15.9 ±7 (range 4.4-31) μmoL / L, NT / TT rate 78.8 ± 7 (range 67.1-91), DD / TT rate 10.5 ± 3.5 (range 4.5-16.4) and DD / NT rate 13.8 ± 5.8 (range 4.9-24.4).

In the prostatitis patient group NT was detected as 116.4 ± 40.5 (range 53.5-281.3) μmoL / L, TT 147.5 ± 50.1 (range 61-326.8) μmoL / L, DD 15.5 ± 8.7 (range 2.7-32.3) μmoL / L, NT / TT rate 79.7 ± 9 (range 66.7-94.7), DD / TT rate 10.1 ± 4 .5 (range 2.6-16.6) and DD /NT rate 13.5 ± 7.2 (range 2.7-24.9).

In the control group NT was detected as 144.1 ± 21.2 (range 106.9-194.2) μmoL / L, TT 191 ± 32.3 (range 133.3-280.4) μmoL / L, DD 23.4 ± 10.1 (range 5-43.1) μmoL / L, NT / TT rate 76.1 ± 98.3 (range 66.2-92.8), DD / TT rate 11.9 ± 4.1 (range 3.5-16.8) and DD / NT rate 16.4 ± 6.9 (range 3.8-25.5).

**Figure 1:**
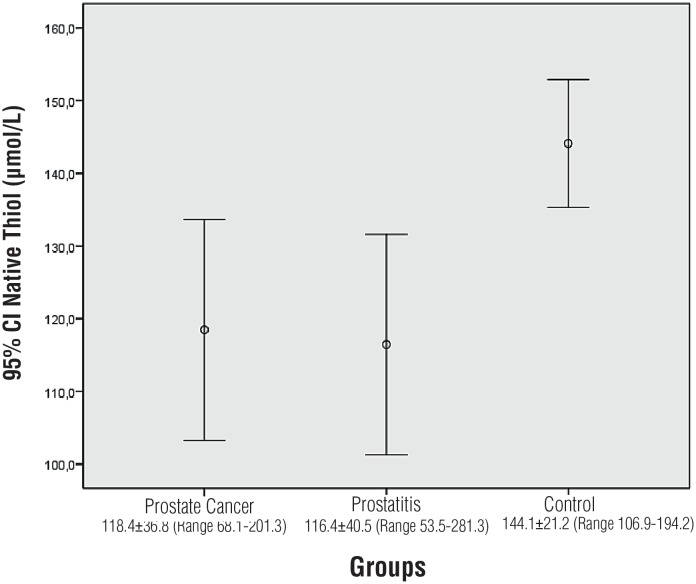
Comparison of native thiol levels.

NT, TT and DD levels were detected to have normal distribution. Significant difference was detected between the three groups in NT levels (p = 0.008) (Table-2). Significant difference was not detected between prostate cancer and prostatitis in the Tukey post-hoc analysis (p = 0.975). Significant difference was detected between the prostate cancer group and the control group (p = 0.027). Significant difference was detected between the prostatitis group and the control group (p = 0.011) (Figure-1).

**Table 2:** Differences between groups.

Variable	Prostate Cancer (n:25)	Prostatitis (n:30)	Control Groups (n:25)	p Value
Native Thiol (μmoL / L)	118.4±36.8	116.4±40.5	144.1±21.2	0.008*
Mean±SD (min-max)	(68.1-201.3)	(53.5-281.3)	(106.9-194.2)	
Total Thiol (μmoL / L)	150.3±45.3	147.5±50.1	191±32.3	0.001*
Mean±SD (min-max)	(87.2-243.9)	(61-326.8)	(133.3-280.4)	
Dynamic Disülfide(μmoL / L)	15.9±7	15.5±8.7	23.4±10.1	0.002*
Mean±SD (min-max)	(4.4-31)	(2.7-32.3)	(5-43.1)	
Native Thiol/TotalThiol μmoL / L	78.8±7	79.7±9	76.1±98.3	0.222
Mean±SD (min-max)	(67.1-91)	(66.7-94.7)	(66.2-92.8)	
Dynamic Disulfide/Total Thiol	10.5±3.5	10.1±4.5	11.9±4.1	0.222
Mean±SD (min-max)	(4.5-16.4)	(2.6-16.6)	(3.5-16.8)	
Dynamic Disulfide/Native Thiol	13.8±5.8	13.5±7.2	16.4±6.9	0.222
Mean±SD (min-max)	(4.9-24.4)	(2.7-24.9)	(3.8-25.5)	

**Figure 2:**
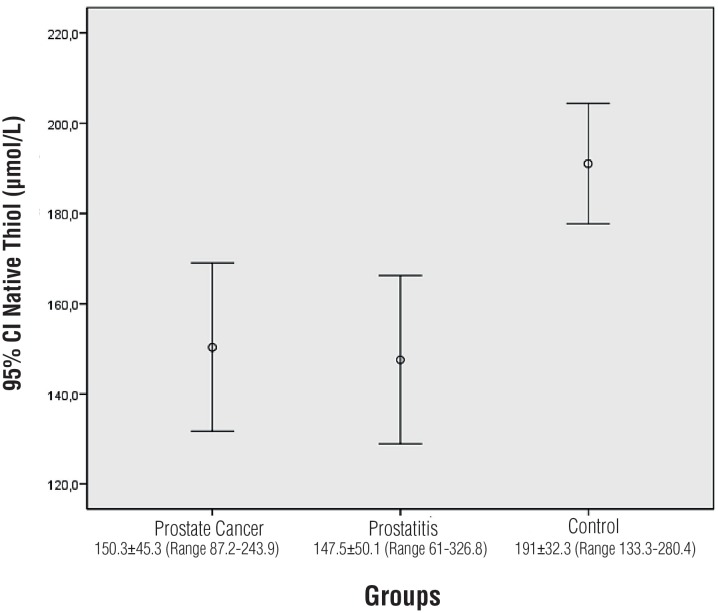
Comparison of total thiol levels.

Statistical difference was detected in terms of TT levels between the prostate cancer, prostatitis and control groups (p = 0.001). Significant difference was not detected between prostate cancer and prostatitis in the Tukey post-hoc analysis (p = 0.970). Significant difference was detected between the prostate cancer group and the control group (p = 0.004). Significant difference was detected between the prostatitis group and the control group (p = 0.001) (Figure-2).

**Figure 3:**
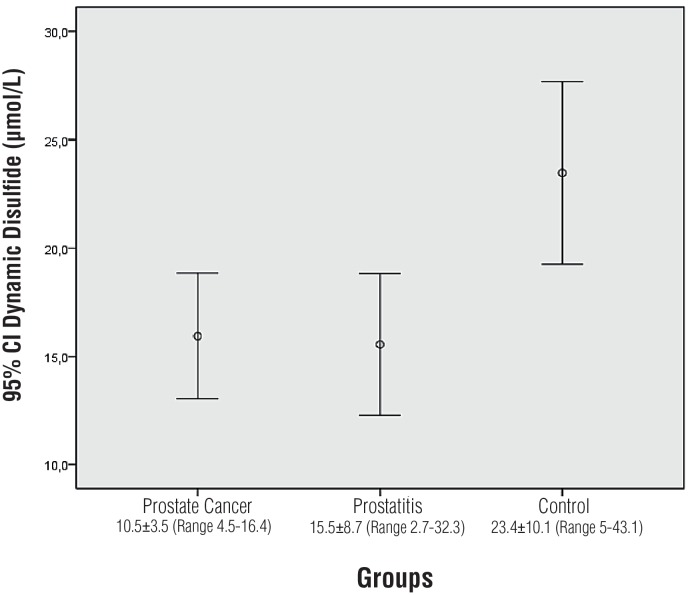
Comparison of Dynamic Disulphide levels.

Statistical difference was detected in terms of DD levels between the prostate cancer, prostatitis and control groups (p = 0.002). Significant difference was not detected between prostate cancer and prostatitis in the Tukey post-hoc analysis (p = 0.986). Significant difference was detected between the prostate cancer group and the control group (p = 0.009). Significant difference was detected between the prostatitis group and the control group (p = 0.004) (Figure-3).

NT / TT, DD / TT and DD / NT ratios were detected to not show normal distribution. No significant difference was detected between the three groups in terms of the NT / TT, DD / TT and DD / NT rates (p = 0.222, p = 0.222, p = 0.222).

When the patients with prostate cancer were separated into risk groups according to t PSA and Gleason score, 2 (8%) had low risk, 8 (32%) had moderate risk and 15 (60%) had high risk. Significant difference was not detected between NT, TT and DD levels within the risk groups (p = 0.742, p = 0.551, p = 0.762). Significant difference was also not detected between NT / TT, DD / TT and DD / NT rates within the risk groups (p = 0.431, p = 0.431, p = 0.431).

## DISCUSSION

In our study we considered that serum NT, TT and DD levels could be a good marker in differentiating patients with high PSA values and normal patients. In this study we aimed to show the change in thiol/disulphide values in two different diseases with acute and chronic progression occurring on the same tissue.

Many studies have been conducted on oxidative stress in urological patients regarding prostate cancer, benign prostatic hyperplasia or prostate inflammation (12–14). Some studies investigated oxidative markers in semen and urine (15). However, there are very few studies regarding thiol / disulphide in the field of urology (16, 17). In this paper, an invasive procedure such as a biopsy has been shown to increase oxidative stress in the prostate tissue (17). Normal oxidative stress markers have been used for a very long time. However, using the current method developed by Dr. Erel and Dr. Neselioglu, plasma dynamic thiol / disulphide homeostasis can be measured with faster, more inexpensive, practical and fully-automatic spectrophotometric examination (10).

Many recent studies have shown disorder in plasma thiol / disulphide homeostasis in the enteropathogenesis of diabetes mellitus, cardiovascular diseases, cancer, rheumatoid arthritis, chronic kidney disease, Parkinson disease and liver diseases (18–21). Therefore, determining dynamic thiol-disulphide homeostasis can provide many valuable information to detect the physiologic and pathologic biochemical process of many diseases. In their study, Erel et al. detected high plasma disulphide values in patients who were smoking, had diabetes, obesity and pneumonia and detected low values in patients with diseases such as bladder cancer, colon cancer, kidney cancer and multiple myeloma. Disulphide values were detected as very low in rapidly growing tumors while as slightly lower than normal in slow-progressing diseases (10). In some studies, it was detected that DD / NT ratios had positive correlation with age while in some studies it was shown to have negative correlation (22, 23). In our study, a clear evaluation was not made on correlation with age because age did not have normal distribution between the groups.

Studies have shown that oxidative stress biomarkers such as thiobarbituric acid reactive substances, total oxidative status, malondialdehyde, plasma nitrite / nitrate levels, lipid peroxide activities increased in prostate cancer patients compared to the control group (24). Some studies have reported decrease in antioxidant enzymes such as catalase, superoxide dismutase containing manganese, superoxide dismutase containing copper and zinc, glutathione peroxidase, disrupted oxidative stress / antioxidant status in prostate cancer patients (25). In another study it was determined that prostate cancer progression and oxidative stress had positive correlation and shown that anti-oxidants such as vitamin E and selenium reduced this risk (26). Thiol / disulphide homeostasis and thiol oxidation have critical importance in protecting cells against detoxification, arranging enzymes and important cellular pathways such as proapoptotic, signal transmission and antiapoptotic signalization (27).

PSA is the most commonly used marker in prostate cancer diagnosis. PSA value, tumor volume and Gleason score are the most important prognostic factors in the course of prostate cancer. High PSA values were present in both groups in our study. PSA level increases rapidly in the event of acute bacterial prostatitis. However, blood values such as CRP, ESR and White Blood Cell also rise.

Prostate cancer is a chronic disease (except for high-risk prostate cancer), PSA values usually rise slowly and can also be detected at extremely high values depending on tumor aggressiveness. In this study, we aimed to show the change in thiol / disulphide values in two different diseases with acute and chronic progression occurring on the same tissue. According to our findings, NT, TT and disulphide values were found to be significantly different in both prostatitis and the prostate cancer compared to the control group. However, a statistically significant difference was not observed when both groups were compared. This shows that both cancer and inflammation trigger a similar oxidative stress on the tissue. Although oxidative stress markers rise suddenly in acute events, this increase takes place gradually in chronic processes. However, this process continues in cancer patients unless treated. In inflammation, the oxidative process returns to normal after the causative infectious event is remedied. Thiols, which are an anti-oxidant structure in the serum, may have decreased upon exposure to severe oxidation because anti-oxidant defense weakens or oxidation increases in prostate cancer or prostatitis patients. Oxidation products more progressed than disulphides were formed as thiols were subject to severe oxidation. As these are usually products of irreversible thiol oxidation, it is considered that disulphides are also low (10).

Earlier studies have not shown the relation between tPSA level and thiol / disulphide values. In our study tPSA values and thiol/disulphide values were compared in both groups. However, although tPSA values were high in both groups, it was detected to be statistically higher in the prostate cancer group. However, no difference was detected between both groups in Thiol / Disulphide ratios. High tPSA values are a direct indicator of prostate tissue damage.

The main limitations of the present study are its retrospective and non-randomized nature. In addition, the number of patients involved is small. These results need to be supported by prospective, randomized studies, and comprehensive patient series.

## CONCLUSIONS

Calculating thiol / disulphide values, which is a new marker, is an easy, inexpensive and reliable method. Serum NT, TT, DD levels in patients with prostatitis and prostate cancer were found significantly lower compared to the control group. This shows that just as inflammation, prostate cancer also increases oxidative stress on tissues. We consider that NT, TT, DD levels measures in serum can be used in the differential diagnosis of these pathologies.

## Declarations

Approval was obtained for this research with the decision of the SANKO University Faculty of Medical Local Ethics Committee dated 29.03.2018 / numbered 2018 / 04 and the study was conducted in compliance with the Helsinki Declaration Rules. All patients participating in the study were informed about the study and their written consent was obtained. The data supporting the results reported in the article are applicable.

The authors declare that they have no competing interests.

We have no funding body in this study. MS did physical examinations and acquired the data of patients. HÇ studied on design and biochemical parameters. MY performed the statistical analysis and interpretation of data drafting of the manuscript. NO worked on critical revision of the manuscript for important intellectual content. All authors read and approved the final manuscript.

## ABBREVIATIONS

NT: Native Thiol
TT: Total Thiol
DD: Dynamic Disulphide
ROT: Reactive Oxygen Types
PSA: Prostate Specific Antigen
tPSA: Total Prostate Specific Antigen
ABP: Acute Bacterial Prostatitis
ESR: Erythrocyte Sedimentation Rate
CRP: C-Reactive Protein
NaBH4: Sodium Borohydride
DTNB: 5,5’-dithio-bis-(2-nitrobenzoic acid))
